# Systematic Investigation on the Swelling Response and Oil Resistance of NBR Using the Prediction Models Determined by the Modified Flory–Huggins Interaction Parameter

**DOI:** 10.3390/polym16192696

**Published:** 2024-09-24

**Authors:** Yiran Jing, Guangyong Liu

**Affiliations:** Key Laboratory of Rubber-Plastics, Ministry of Education, Qingdao University of Science & Technology, Qingdao 266042, China; 17863957197@163.com

**Keywords:** three-dimensional solubility parameter, Flory–Huggins interaction parameter, prediction model, swelling response, NBR

## Abstract

The equilibrium swelling test was employed to determine the swelling response of Nitrile Butadiene Rubber (NBR) with various acrylonitrile (ACN) contents, and the three-dimensional solubility parameter (HSP) and modified Flory–Huggins interaction parameter (χ_HSP_) were used to establish the prediction model of the oil-resistant property. The results indicate that the energy difference (Ra) between NBR and solvents calculated by HSP values can be correlated with the swelling response qualitatively with an inversed “S-shape”, and high swelling response occurs at Ra < 8 MPa^1/2^ for NBR. For the purpose of establishing the prediction model, the new modified χ_HSP_ value has been calculated and fitted with the swelling response using exponential and logarithmic fittings, respectively. Two prediction models considering all the possible influencing factors have been obtained to determine the swelling response and oil resistance of NBR-based rubber products in bio-fuels, represented by the bio-diesel and IRM 903 test oil in this work. The swelling response of NBR can be evaluated precisely, and high swelling regions can be predicted and avoided in the new emerging fuels through the prediction models. Thus, the oil resistance of NBR-based rubber products, such as seals, holes and gaskets can be well predicted now.

## 1. Introduction

Nitrile butadiene rubber (NBR) is an unsaturated polar polymer which is copolymerized with butadiene and acrylonitrile monomers [1,2]. The polarity and unsaturation of NBR are related to the acrylonitrile content. With the increase in acrylonitrile content, the molecular polarity, intermolecular force and cohesion energy density increase, leading to the improvement of the anti-static property and non-polar oil resistance [3,4,5]. Due to the good resistance to non-polar media (gasoline, kerosene, etc.), NBR is mainly used in a variety of oil resistant and antistatic products, such as oil pipes, sealing strips, textile rollers, and so on [6,7,8]. Therefore, the study of the diffusion and swelling behaviors of NBR in different media becomes necessary.

Hildebrand and Scott [9] first introduced the concept of a solubility parameter (δ) to characterize the thermodynamic behavior of non-electrolytes in binary systems, which can be used to predict the polarity, solvent resistance, and polymer–polymer compatibility of substances.

(1) $$\delta ={\left[\frac{E_{coh}}{V_{mol}}\right]}^{1/2}$$

where δ is the solubility parameter with the unit of MPa^1/2^, E_coh_ is the cohesive energy of a substance, representing the amount of energy required to hold one mole of molecules together, and V_mol_ is the molar volume.

This formula has limitations in applications when there are polar interactions because the dissolution becomes more complex (even the solvation process occurs when the solubility parameters of the solvent and polymer are quite different) [10,11,12]. Based on Hildebrand’s regular solution theory, Hansen extended the solubility parameter to polar and conjugated systems, and established a three-dimensional solubility parameter system, abbreviated as HSP [13]. The cohesion energy of a substance is considered to be the sum of various interaction forces between molecules, including the van der Waals force, covalent bond, hydrogen bond, ionic bond, electrostatic action, the permanent dipole moment and the induced dipole moment. Hansen divided the cohesion energy of a substance into the sum of the contribution values of three components: the dispersion force (E_d_), the polarity force (Ep) and the hydrogen bond force (E_h_) [14,15]. By extending the solubility parameter to a polar system and an association system, a three-dimensional solubility parameter system is established as follows:

(2) $$\delta_{d}={\left(\frac{E_{d}}{V}\right)}^{\frac{1}{2}},\;\;\delta_{p}={\left(\frac{E_{p}}{V}\right)}^{\frac{1}{2}},\;\;\delta_{h}={\left(\frac{E_{h}}{V}\right)}^{\frac{1}{2}}$$

(3) $$\delta_{t}=\delta_{d}^{2}+\delta_{p}^{2}+\delta_{h}^{2}$$

where δ_t_ is the Hildebrand solubility parameter, δ_d_, δ_p_, and δ_h_ correspond to the three partial solubility parameters.

It provides an easy method for predicting the compatibility of polymer blends, selecting solvents, and predicting polymer–solvent interactions, which has been widely used in polymer solutions [16,17,18].

The concept of HSP can be more intuitively reflected in a three-dimensional spatial graph: δd, δp, and δh are the three coordinate axes with the polymer as the spheric center. The interaction strength between polymer and solvent is taken as the radius (R_0_) to obtain a sphere named as the solubility sphere of polymer. The interaction between polymer and solvent can be expressed in terms of their spatial distance Ra (energy difference). The solvent can dissolve or swell the polymer quickly when it is inside of the solubility sphere (Ra < R_0_) [19].

(4) $$Ra={\left[a{\left(\delta_{d}^{P}-\delta_{d}^{S}\right)}^{2}+{\left(\delta_{p}^{P}-\delta_{p}^{S}\right)}^{2}+{\left(\delta_{h}^{P}-\delta_{h}^{S}\right)}^{2}\right]}^{1/2}$$

where δi (i = d, p, h) for each of the three components of the HSP values, and the superscripts P and S represent the polymer and solvent, respectively.

The solubility parameter is an important thermodynamic parameter of polymers, which can predict the polarity of a substance, the solvent resistance, and the polymer–polymer compatibility [20,21,22]. The solubility parameter of a low molecular solvent can be obtained directly from the enthalpy of evaporation [23,24]. Due to the high molecular weight of polymers, as well as the possible existence of the macromolecular crystallization and degradation and other factors, the solubility parameter of polymers cannot be directly obtained by evaporation. Therefore, indirect methods have to be used to determine the solubility parameter, including the optical refractive index method [25], the turbidimetric titration method [26], gas chromatography [27], the intrinsic viscosity method [28] and the equilibrium swelling method [29,30,31].

The ACN content determines the key properties of NBR vulcanizates, for example, the oil resistance and low-temperature properties. The ACN content of NBR determines its polarity, which in turn determines the resistance to apolar oils and greases. Thus, due to the large range of different compositions available, adequate NBR grades can be selected for many applications. In this work, three different NBR vulcanizates with incremental ACN contents are selected for determining the swelling responses in various solvents through the equilibrium swelling tests. The HSP values and the modified χ_HSP_ parameters are calculated to establish the prediction models, which are expected to predict the swelling responses and oil resistance of NBR-based rubber products in newly emerged bio-fuels.

## 2. Experimental Section

### 2.1. Materials

The Nitrile butadiene rubber with the ACN contents of 34 wt% (NBR34), 39 wt% (NBR39) and 44 wt% (NBR44), respectively, are supplied by ARLANXEO (The Hague, The Netherlands). The crosslinking system composing of 2, 4-di-tert-butylisopropylbenzene peroxide (Luperox F40) and triallyl isocyanurate (TAIC) are purchased from Arkema (Paris, France). Organic solvents (analytical reagent) used for the swelling tests, including alkanes, aromatics, ethers, esters, ketones, nitriles and amides are commercially available industrial products.

### 2.2. Equilibrium Swelling Test

Only the crosslinking system (Luperox F40 6 phr, TAIC 2 phr) was used to reduce the influence of other factors. NBR was mixed with the crosslinking system directly using a two-roll mill (BOLON Precision Testing Machines Co. Ltd., BL-6175, Dongguan, China) at 50 °C with a roller speed ratio of 1:1.2. Then, uniformly dispersed NBR compounds were obtained. The vulcanized rubber sheets of a 2 mm thickness were obtained by a heated curing press at 180 °C and t90 + 5 min as the curing time determined by the Moving Die Rheometer (MDR2000, Alpha Technologies, Bellingham, WA, USA).

Circular samples with the diameter of 12 mm were cut from the vulcanized sheets for the swelling tests. Five samples were selected for each test to reduce the weighing error. These samples were first weighed for the initial weight, then immersed in the selected solvents for the swelling tests. At specifical intervals, the samples were taken out, and extra solvent on the surface was removed quickly with filter paper. Then, the samples were weighed immediately. After weighing, the samples were placed back into the original test bottles. Such operations were conducted several times until reaching equilibrium swelling and then the samples were weighed again to obtain the final weight. The equilibrium swelling tests were carried out to calculate the swelling ratio (q) of the NBR samples in the well-selected solvents, as shown in Table 1.

The swelling process in detail can be found in our previous study [32], and q is determined as follows:

(5) $$q=\frac{W_{S}/\rho_{S}}{W_{R}/\rho_{R}}$$

where W_S_ is the mass uptake of the solvent at an equilibrium state, W_R_ is the initial mass of the rubber sample before swelling, ρ_S_ and ρ_R_ are the densities of the solvent and rubber sample, respectively. Herein, q can be defined as the volume of the absorbed solvent per unit volume of the rubber sample in units of mL/cm^3^.

## 3. Results and Discussion

### 3.1. Correlation of Swelling Ratio with Solubility Parameter

**Figure 1:**
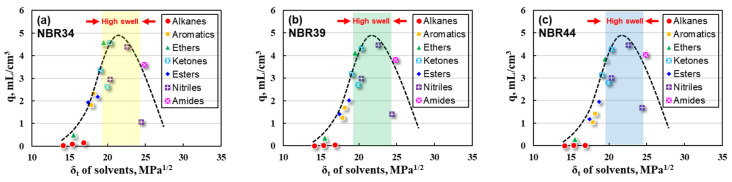
Plots of q values for NBR with δ_t_ values for solvent (**a**) NBR34, (**b**) NBR39, and (**c**) NBR44.

The swelling ratio (q) of NBR varies greatly in different solvents, which may be related to the one-dimensional solubility parameter (δ_t_) of the solvent. In order to better compare this relationship, q values of three NBR vulcanizates (NBR34, NBR 39, and NBR44) were preliminarily correlated with the δ_t_ values of solvents, as shown in Figure 1.

It can be clearly seen that the q values of NBR vulcanizates increase first and then decrease with the increase in the δ_t_ values of the solvents, passing through high swell regions corresponding to the range of δ_t_ values from 20 to 25 MPa^1/2^ (dashed line in Figure 1). Therefore, the δ_t_ value corresponding to the maximum q value can be regarded as the one-dimensional solubility parameter (δ_t_) of NBR, which is estimated within the range of 21~24 MPa^1/2^. However, it is impossible to distinguish the specific δ_t_ values for the three different NBR samples, i.e., NBR34, NBR39, and NBR44.

For this reason, a computer program was employed to calculate the HSP values of NBR by inputting the swelling data. The swelling ratios (q) of NBR in these solvents were used directly as input data into the computer program to determine the specific three-dimensional solubility parameters. The range of the swelling ratio was from 0 to 5 mL/cm^3^ and, therefore, the calculation routine of the program assumed the intermediate value of ca 2.5 mL/cm^3^ as the starting point and the boundary condition between a high and low swelling response. The program analyses and analog computes the swelling ratio, and outputs HSP values along with HSP graphs of NBR34, NBR39, and NBR44, as shown in Table 2. The computational procedure of the program has been described in detail in our previous study [28].

The dispersion force component (δ_d_) of NBR34, NBR39, and NBR44 rubber is basically the same, the difference in the hydrogen bond force component (δ_h_) is small, and the polar force component (δ_p_) is quite different, which is mainly caused by the incremental ACN content.

In high swelling regions, however, there are still some solvents, such as acetone and DMF, showing relatively low swelling responses even if their δ_t_ values are within the same range of 21~24 MPa^1/2^. This may be because the δ_p_ and δ_h_ values of these two solvents are quite different from those of NBR.

**Figure 2:**
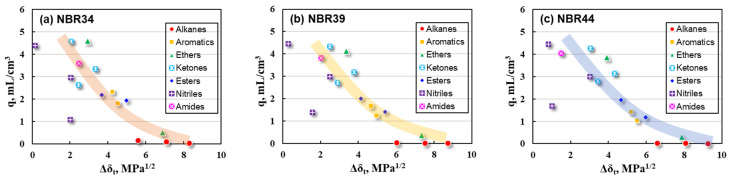
Correlation of q with Δδ_t_ for NBR vulcanizates (**a**) NBR34, (**b**) NBR39, and (**c**) NBR44.

Further comparison of the q value of NBR with the Δδ_t_ between NBR and solvent was studied as shown in Figure 2.

The color bands are added into Figure 2 to illustrate the possible trends in q with Δδ_t_ values. There is a rough relationship between q and Δδ_t_ for NBR vulcanizates, indicating that a higher Δδ_t_ value relates to a lower q value, and vice versa. However, there are still many discrete points in this correlation, especially for nitrile solvents exhibiting a small q value even if the Δδ_t_ is low. The Δδ_t_ cannot explain such a phenomenon, and hence, it is necessary to further use the HSP values to solve this problem.

**Figure 3:**
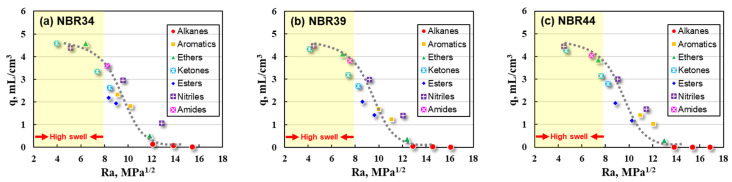
Correlation of q values with Ra for NBR vulcanizates (**a**) NBR34 (**b**) NBR39 (**c**) NBR44.

Ra values calculated by HSP values were used to correlate the swelling ratios of NBR vulcanizates and the results were shown in Figure 3.

The q values of NBR34, NBR39, and NBR44 vulcanizates increase with the decrease in Ra values, showing inversed S-shapes (dashed lines in Figure 3). The swell response is in the high swell region (yellow band in Figure 3) when the Ra is less than 8 MPa^1/2^; on the contrary, the swelling ratio becomes lower at a higher Ra > 8 MPa^1/2^, and it becomes quite small especially when the Ra is greater than 12 MPa^1/2^. Better compatibility between the NBR and solvent contributes to a higher swelling response which can be reflected in a smaller Ra value as well. Further observation of Figure 3 shows that there is no obvious discrete point on the q~Ra curve, that is, the correlation of q with Ra is more acceptable compared to the q~Δδ_t_ curve. NBR is usually used to make protective gloves, seals, holes and gaskets, and thus, the liquid medium resistance is very important for NBR-based products. The Ra value shows better advantages in predicting the oil resistance of NBR vulcanizates and avoiding the potential risks of liquid leakage.

However, the inversed “S-shape” curve is still difficult to express precisely or be quantitatively fitted by a mathematical equation, which makes it impossible for us to establish the prediction model of the swelling response for an NBR vulcanizate.

### 3.2. Flory–Huggins Interaction Parameters of NBR/Solvent

The increasing use of new fuels and oils such as bio-diesel and bio-gasoline and their effects on rubber materials have aroused widespread concern in the industry. NBR-based seals and gloves are often in contact with organic liquids with great differences in polarity, and the interaction between NBR and different liquids should be fully considered. The three-dimensional solubility parameters and Flory–Huggins interaction parameters (χ) provide a concise method for studying the interactions existing in NBR swelling systems.

The χ parameter was determined originally based on the corresponding state theory (CST), which was used to calculate the χ values of non-polar systems (non-polar rubber and non-polar solvents) and to characterize the thermodynamic state of rubber macromolecular solutions [33,34]. The equation is shown as follows:

(6) $$\chi =\chi_{H}+\chi_{S}$$

where χ_S_ is the entropy correction term with a constant of 0.34 for a strictly nonpolar system. χ_H_ refers to the contribution of the enthalpy change which can be calculated by the δ_t_ values of the rubber and solvent:

(7) $$\chi_{H}=\frac{V}{RT}{\left(\delta_{p}-\delta_{s}\right)}^{2}$$

Therefore, for the non-polar rubber solution system, the following calculation formula can be obtained:

(8) $$\chi =0.34+\frac{V_{m}}{RT}{\left(\delta_{p}-\delta_{s}\right)}^{2}$$

where V_m_ is the molar volume of a small molecule solvent, R is the ideal gas constant, T is the absolute temperature, and δp and δs are one-dimensional solubility parameters of the rubber and solvent, respectively.

According to Flory [35,36,37], a provided condition under which a rubber and a solvent are expected to be completely miscible through the entire composition range is the following,

(9) $$\chi <\frac{1}{2}{\left[1+{\left(\frac{V_{s}}{V_{p}}\right)}^{1/2}\right]}^{2}$$

Therefore, it can be inferred that a critical condition or a critical interaction parameter (χ_C_) for rubber–solvent miscibility is,

(10) $$\chi_{c}=\frac{1}{2}{\left[1+{\left(\frac{V_{s}}{V_{p}}\right)}^{1/2}\right]}^{2}$$

where V_P_ and V_S_ are the molar volumes of rubber macromolecules and a small molecule solvent, respectively.

Comparing V_P_ and V_S_ values, it can be considered that V_S_/V_P_ tends to be zero. Therefore, the condition of a rubber dissolving in a solvent is χ < 0.5 for the non-polar system. The δp and δs values in Equation (8) need to be very close to each other to meet such a condition that the rubber is sufficiently dissolved in the solvent.

However, Equation (8) shows obvious limitations in describing the polar rubber solution system because it does not consider the contributions of the polar and the hydrogen bond forces to the rubber dissolution process. It is attempted to solve this problem by using a modified Flory–Huggins interaction parameter (χ_HSP_) that can be calculated by the HSP values for the polar rubber solution system, as follows.

(11) $$\chi_{\mathrm{HSP}}=\frac{V_{S}}{4RT}\left[4{\left(\delta_{d}^{P}-\delta_{d}^{S}\right)}^{2}+{\left(\delta_{p}^{P}-\delta_{p}^{S}\right)}^{2}+{\left(\delta_{h}^{P}-\delta_{h}^{S}\right)}^{2}\right]$$

(12) $$\chi_{HSP}=\frac{V_{S}}{4RT}Ra^{2}$$

Equation (12) for calculating the χ_HSP_ value includes the contributions from the dispersion force, the polar force and the hydrogen bond force and takes into account the influences of molar volume and temperature. For real rubber solutions or swelling systems, special interactions such as polarity and hydrogen bonding contribute more to the χ value, resulting in some unpredictable miscibility or swelling properties. Therefore, in most cases, Equation (12) is more suitable for the evaluation of the compatibility between rubber and solvent when handling the swelling and dissolving processes [38,39].

### 3.3. Prediction Model for the Swelling Response of NBR

**Figure 4:**
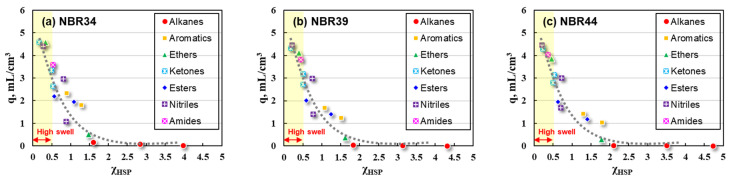
Plots of q with χ_HSP_ values for (**a**) NBR34, (**b**) NBR39, and (**c**) NBR44.

In many practical applications, NBR-based rubber products are often exposed to a variety of polar fluids, and the prediction of the swelling properties in various solvents or solvent mixtures becomes particularly important. There are many factors affecting the swelling behaviors of NBR, including the structures of the NBR and the liquid, the size of the liquid, and the temperature. The new χ_HSP_ value provides an effective way to construct the prediction model of the swelling properties by fully considering all the possible influencing factors. Therefore, it is attempted to correlate q with the χ_HSP_ value for NBR vulcanizate, and the results are shown in Figure 4.

It can be seen from Figure 4 that q increases with the decrease of the χ_HSP_ value (dashed line in Figure 4). Assuming q = 2.5 mL/cm^3^ as the boundary condition, the high swelling region (yellow band in Figure 4) corresponds to the χ_HSP_ value being less than 0.5. This result agrees well with the theory based on the Hildebrand regular solution model, which holds that for the actual rubber swelling system, the swelling becomes more complex, but the χ value must be less than 0.5 for the rubber–solvent system with a completely miscible or high swelling state.

**Figure 5:**
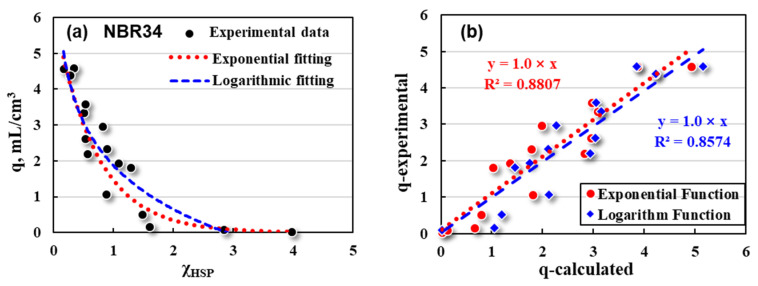
Mathematical fittings for NBR34’s (**a**) q and χ_HSP_, and its (**b**) q values between experimental and calculation data.

Furthermore, mathematical fitting between q and the χ_HSP_ value was made for the purpose of predicting the swelling response of NBR quantitatively by theoretical calculation. It can be preliminarily judged from Figure 4 that there might be an exponential or logarithmic relationship between q and χ_HSP_, as shown in Figure 5. Similar mathematical fittings have been adopted to Fluororubber (FKM) and Hydrogenated Nitrile rubber (HNBR) due to the relatively high degree of fittings in our previous study [28].

As shown in Figure 5a, exponential (red dotted line) and logarithmic (blue dashed line) fittings are performed on the relationships of q (black dots) with the χ_HSP_ as follows:

(13) $$\text{Exponential: q}=6.2\times e^{\left(-1.4\times \chi_{HSP}\right)}\;R^{2}=0.8789$$

(14) $$\text{Logarithm: q}=1.9-1.8\times \ln\left(\chi_{HSP}\right)\;R^{2}=0.8574$$

Both exponential fitting and logarithmic fitting show relative high degree of fittings, which are quite different from the linear relationship of non-polar styrene-butadiene rubber (SSBR) [40,41]. One of the main purposes of the theoretical calculation of χ_HSP_ is to predict the swelling response and oil resistance of NBR parts in different fluids.

Therefore, the swelling ratios (q-calculated) of NBR34 in each solvent were calculated quantitatively by using exponential Equation (13) and logarithmic Equation (14), respectively, and compared with the experimental ones (q-experimental), as shown in Figure 5b. q-calculated and q-experimental values have some discreteness, but basically show a good linear relationship. The fitting degree of the exponential relationship is slightly higher than that of logarithmic fitting. It can be deduced that the logarithmic fitting may have negative q values when χ_HSP_ values are large (poor compatibility). Such a phenomenon is called swelling shrinkage, which is the case of the oil-resistant rubber products in some extreme fluids.

**Figure 6:**
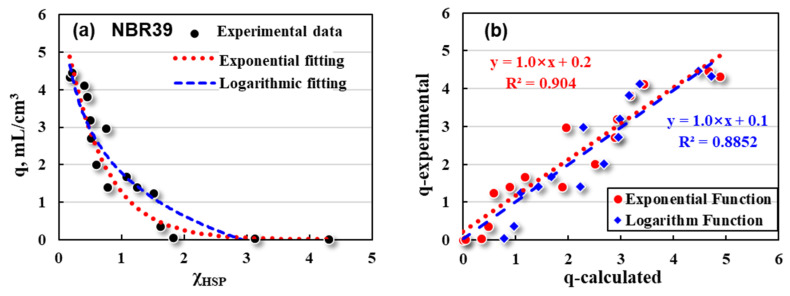
Mathematical fittings for NBR39’s (**a**) q and χ_HSP_, and its (**b**) q values between experimental and calculation data.

**Figure 7:**
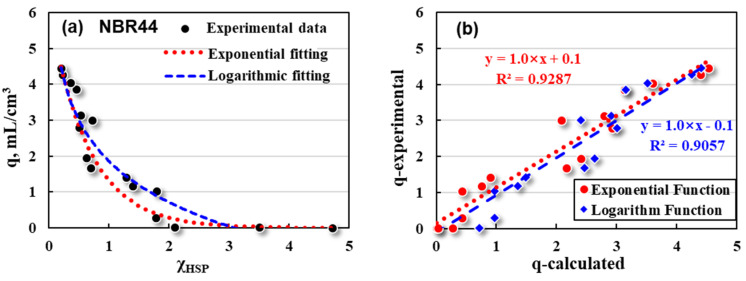
Mathematical fittings for NBR44’s (**a**) q and χ_HSP_, and its (**b**) q values between experimental and calculation data.

Then, exponential and logarithmic fittings of q~χ_HSP_ were made for NBR39 and NBR44, respectively, and the swelling ratios were calculated theoretically. The results are shown in Figure 6 and Figure 7.

Exponential (red dotted line) and logarithmic (blue dashed line) fittings between the q (black dots) and χ_HSP_ for NBR39 are described as follows:

(15) $$\text{Exponential: q}=6.5\times e^{\left(-1.6\times \chi_{HSP}\right)}\;R^{2}=0.9022$$

(16) $$\text{Logarithm: q}=1.8-1.7\times \ln\left(\chi_{HSP}\right)\;R^{2}=0.8852$$

Exponential (red dotted line) and logarithmic (blue dashed line) fittings between the q (black dots) and χ_HSP_ for NBR44 are described as follows:

(17) $$\text{Exponential: q}=6.2\times e^{\left(-1.5\times \chi_{HSP}\right)}\;R^{2}=0.9276$$

(18) $$\text{Logarithm: q}=1.9-1.6\times \ln\left(\chi_{HSP}\right)\;R^{2}=0.9057$$

It can be seen that for NBR39 and NBR44, both exponential and logarithmic fittings show a high degree of fittings. Similarly, the swelling ratios (q-calculated) of NBR39 and NBR44 in each solvent were calculated mathematically and compared with the experimental ones (q-experimental). The results are shown in Figure 6b and Figure 7b. Both the calculated and the experimental q values are discrete to a certain extent, but basically show a good linear relationship. In short, exponential and logarithmic fittings are expected to be suitable for the basic prediction models in evaluating the swelling responses of NBR-based rubber products.

### 3.4. Basic Prediction Models of Swelling Responses

As discussed above, there are exponential and logarithmic relations between q~χ_HSP_. For the three exponential relations, the pre-exponential factors are in the range of 6.2 to 6.5 and the exponential factors are 1.4~1.6. For the three logarithmic relations, the addition factors are in the range of 1.6 to 1.8 and the logarithmic factors are 1.8~1.9. Herein, two general prediction models can be established preliminarily as follows:

(19) $$\text{Exponential: q}=6.3a\times e^{\left(-1.5m\times \chi_{HSP}\right)}$$

(20) $$\text{Logarithm: q}=1.8b-1.8n\times \ln\left(\chi_{HSP}\right)$$

where a and m represent the correction coefficients of the pre-factors for an exponential model, and n and b are the correction coefficients of pre-factors for a logarithmic model, respectively.

It has been discussed above that the swelling ratio of rubber is not only related to the Flory–Huggins interaction parameter (χ_HSP_), but also to the crosslinking density (ν), swelling temperature, filler dosage, and other factors. The temperature and molar volume (Vmol) of the solvent have been considered in the calculation of the χ_HSP_ value, and the crosslink density and filler content need to be considered in the prediction model as well.

The chemical crosslink density (ν_c_) can be determined by the equilibrium swelling test with the aid of the Flory–Huggins theory. Fillers (such as carbon black and silica) have a positive correlation effect on the total crosslink density, which in fact mainly increases the physical crosslink density of rubber (ν_p_). Therefore, the crosslink density needs to be taken into account when establishing a prediction model of rubber swelling. The crosslinking network of rubber is extremely complicated, especially in that the number of the crosslinking points is difficult to be quantified. According to Flory–Rehhner theory, the crosslink density (ν) is related to the volume fraction (v_r_) of rubber, which directly affects the maximum swelling ratio (q) instead of the variation trend between q~χ_HSP_.

Therefore, the modified prediction models of NBR can be obtained as follows:

(21) $$\text{Exponential: q}=6.3a\times e^{\left(-1.5m\times \chi_{HSP}\right)}+\alpha \nu$$

(22) $$\text{Logarithm: q}=1.8b-1.8n\times \ln\left(\chi_{HSP}\right)+\beta \nu$$

where ν is the crosslink density, α and β are correction coefficients based on the crosslink density (chemical and physical crosslink densities).

Actually, these correction coefficients, including a, m, b, and n are related to the swelling system with certain relationships.

### 3.5. Application of Prediction Model in Oil Resistance of NBR

The swelling behavior and oil resistance of NBR-based products in new emerging bio-fuels, bio-gasolines and bio-diesels for example, have become important research topics in both academic and industrial fields. Some fuel mixtures containing small amounts of alcohol, such as ethanol gasoline, have a serious effect on the swelling responses of rubber parts. NBR products are widely used in such aspects, hence, it is very important to determine the swelling responses of NBR in these new liquids.

Bio-diesel is a kind of diesel with different components based on the plant raw materials used, which is considered to be low viscosity, low toxicity and non-flammable environmental diesel. Therefore, bio-diesel is an ideal alternative to petroleum diesel as a fuel source. IRM 903 (ASTM No. 3) is a test oil commonly used in laboratories to evaluate the oil resistance of polar rubber products.

New bio-fuels are configured, respectively, by the bio-diesel and IRM 903 test oil supplemented with anhydrous ethanol, and the oil resistance of NBR in such fuels is explored. One of the applications of χ_HSP_ is to predict the swelling behavior or oil resistance of rubber products in new fuels, especially in extreme environments, for example, at very high and low temperatures. IRM 903 and bio-diesel are mixtures with a carbon atom composition similar to that of the “naphtha high-flash” substance. The HSP values of the two fuels are determined according to the previous work as shown in Table 3.

The HSP values follow the linear additive property, and thus the HSP values of the fluid mixtures, IRM 903/Ethanol and bio-diesel/Ethanol, can be calculated as follows:

(23) $$\delta_{i,mix}=\varphi_{1}\delta_{i,1}+\varphi_{2}\delta_{i,2}$$

where δ_i,mix_ represents the δ_d,mix_, δ_p,mix_, and δ_h,mix_ values of each component, respectively. φ_1_ and φ_2_ are the volume fractions of components 1 and 2, respectively.

**Figure 8:**
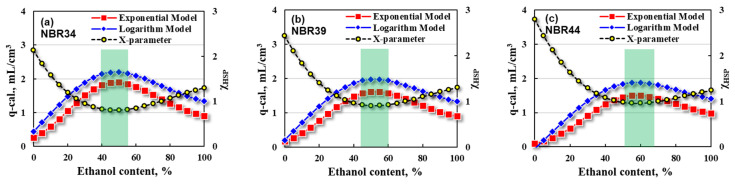
Correlations of q-cal. with ethanol contents in bio-diesel/ethanol blends of (**a**) NBR34, (**b**) NBR39, and (**c**) NBR44.

From the point of view of reducing costs and improving exhaust emissions, organic solvents such as ethanol are often added into bio-diesel or bio-gasoline fuels. For these bio-fuel blends, however, their composition and HSP values will be changed accordingly, which has unforeseen effects on the swelling properties of rubber, and this unforeseeable effect may become more serious especially under high-temperature conditions. For this reason, the swelling responses of NBR in these bio-fuel blends have been calculated by using the prediction models obtained from Equations (21) and (22). Herein, the three NBR vulcanizates, NBR34, NBR39, and NBR44, are fixed with the same crosslink density and other possible factors so as to make the four correction coefficients (a, b, m, and n) equal 1 and the other two crosslinking-related correction coefficients (α, β) equal 0. The swelling ratios (q-cal.) predicted by the prediction models are firstly correlated with the ethanol contents for NBR vulcanizates, as shown in Figure 8.

It can be seen from the bio-diesel/ethanol blends in Figure 8 that the q-cal values obtained by the exponential model (red square) are slightly lower than those calculated by the logarithm model (blue diamond). In addition, with the increase in ethanol content, the q-cal value increases first and then decreases, passing through a maximum swelling region. The corresponding χ_HSP_ value shows the opposite trend, that is, the χ_HSP_ value decreases first and then increases with the increasing ethanol content. The phenomenon is similar but the composition of the bio-diesel/ethanol blends is different when the maximum swelling region appears for the three NBR vulcanizates. The maximum swelling regions correspond to the ethanol contents of 40~55 vol %, 45~60 vol %, and 50~70 vol% for NBR34, NBR39, and NBR44, respectively.

**Figure 9:**
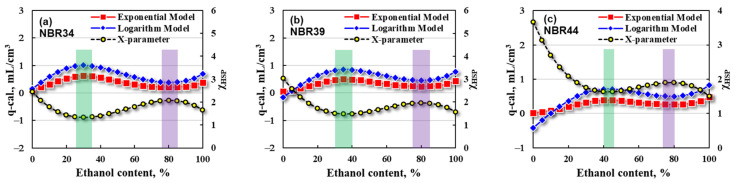
Correlations of q-cal. with ethanol content in IRM903/ethanol blends of (**a**) NBR34, (**b**) NBR39, and (**c**) NBR44.

Further calculations are made using the prediction models for the IRM903/ethanol blends to determine the relationship between the q-cal and ethanol content, as shown in Figure 9.

For the IRM903/ethanol blends, the q-cal values determined by the exponential model (red square) are slightly lower than those obtained by the logarithm model (blue diamond) when the ethanol contents are higher than the three critical values, 10 vol%, 15 vol%, and 20 vol%, respectively, for NBR34, NBR39, and NBR44. However, there exist negative q-cal. values determined from the logarithm model when the ethanol content is less than 10 vol% for NBR34, 15 vol% for NBR39, and 25 vol% for NBR44, respectively. Such a phenomenon is known as shrinkage, that is, the swelling ratio is less than zero, which is often encountered in oil-resistant rubber hose products. The shrinkage becomes more evident while the compatibility between rubber and liquid is worse. In addition, there exists a high swelling region (max q-cal.) and a low swelling region (min q-cal.) with the increase in ethanol content, and the corresponding χ_HSP_ value shows an opposite trend. It can be further found that the low swelling region becomes less noticeable for the NBR with a higher ACN content, and even a swelling platform comes out for the NBR44 which has the highest ACN content.

Additionally, the high swelling and low swelling regions of NBR vulcanizates correspond to different ethanol contents of IRM903/ethanol blends, which have been depicted in Figure 9 (green and purple shadows). Taking NBR34 as an example, a high swelling region appears at the ethanol content of 40~50 vol% and a low swelling region appears at 80~90 vol%. The high swelling region shifts to the direction of a higher ethanol content when the ACN content of NBR increases, or the polarity of NBR is higher.

**Figure 10:**
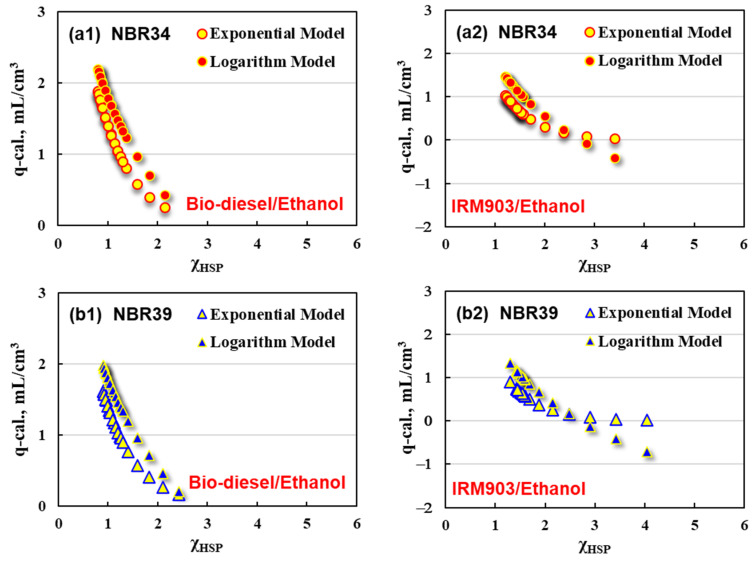

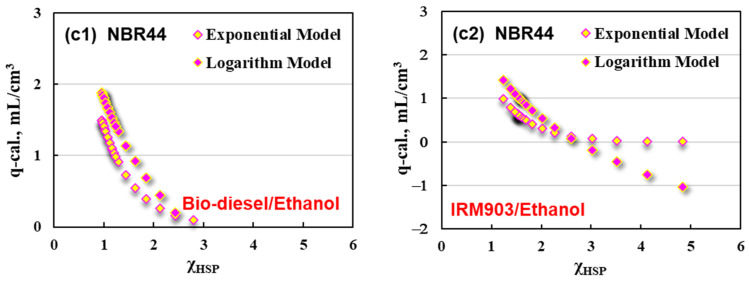
Correlation of q-cal. with χ_HSP_ for (**a1**) NBR34 in bio-diesel/ethanol, (**a2**) NBR34 in IRM903/ethanol, (**b1**) NBR39 in bio-diesel/ethanol, (**b2**) NBR39 in IRM903/ethanol, (**c1**) NBR44 in bio-diesel/ethanol, and (**c2**) NBR44 in IRM903/ethanol.

The q-cal. values determined by the exponential and logarithmic models are further correlated with χ_HSP_ values for bio-diesel/ethanol and IRM903/ethanol blends, respectively. The results are shown in Figure 10.

It can be observed for the bio-diesel/ethanol blends (Figure 10a1,b1,c1) that q-cal. values determined by the exponential model present a being slightly lower than the q-cal. values obtained by the logarithm model. Additionally, the NBR with the lowest ACN content displays the highest swelling response (highest q-cal. value).

It becomes more complicated for the IRM903/ethanol blends in regard to the relationship between the q-cal. and χ_HSP_ value, as shown in Figure 10a2,b2,c2. The q-cal. value decreases with the increasing χ_HSP_ value, but the q-cal. ~χ_HSP_ curves obtained from exponential and logarithm models show an intersection point (χ_IN_) at a certain χ_HSP_ value. q-cal. values calculated by the logarithm model are higher than those of the exponential model when χ_HSP_ is less than χ_IN_, and vice versa. What is interesting is that χ_IN_ is 2.5, the same value for all the NBR vulcanizates, which may provide important theoretical guidance for predicting the oil resistance of NBR-based rubber products in practical applications.

## 4. Conclusions

The increasing use of new bio-fuels, like bio-gasoline and bio-diesel have imposed more challenges on rubber materials. Therefore, the prediction of the swelling response and oil resistance of rubber products in such bio-fuels has become very desirable. Based on this, prediction models of the swelling responses for NBR vulcanizates have been established using modified χ_HSP_ parameters. The swelling ratios increase with the decrease in χ_HSP_ values following both exponential and logarithmic relationships. The prediction models are developed for the purpose of predicting and quantitative evaluating the swelling responses of NBR vulcanizates in bio-fuels which, in this work, are represented by bio-diesel/ethanol and IRM903/ethanol, respectively. Different phenomena, including dilation and shrinkage after swelling, have been predicted for NBR-based rubber products, which are frequently used in industrial and automotive applications, such as in bio-fuels, hydraulic fluids, and lubricants. Potentially, the prediction models obtained in this work can provide a concise way to pre-screen possible elastomers to meet the oil-resistant performance criterion, especially in some extreme application environments.

## Figures and Tables

**Table 1:** The HSP values of the solvents and the swelling ratios of the NBR vulcanizates.

Solvents		δ_d_, MPa^1/2^	δ_p_, MPa^1/2^	δ_h_, MPa^1/2^	q, NBR34	q, NBR39	q, NBR44
Alkanes	Isooctane	14.10	0.00	0.00	0.03	0.01	0.01
	Heptane	15.30	0.00	0.00	0.09	0.03	0.02
	Cyclohexane	16.80	0.00	0.20	0.16	0.05	0.02
Aromatics	Ethylbenzene	17.80	0.60	1.40	1.81	1.24	1.03
	Toluene	18.00	1.40	2.00	2.33	1.68	1.42
Ethers	Diethyl ether	14.50	2.90	4.60	0.51	0.36	0.29
	THF	16.80	5.70	8.00	4.59	4.12	3.86
Esters	Butyl acetate	15.60	6.20	4.90	3.35	3.19	3.14
	Methyl acetate	15.50	7.20	7.60	2.63	2.71	2.79
Ketones	2-butanone	16.00	9.00	5.10	4.58	4.33	4.27
	Acetone	15.50	10.40	7.00	1.94	1.41	1.17
	Cyclohexanone	17.80	8.40	5.10	2.19	2.01	1.95
Nitriles	Butyronitrile	15.30	12.40	5.10	2.96	2.98	3
	Benzonitrile	18.80	12.00	3.30	4.39	4.46	4.45
	Acetonitrile	15.30	18.00	6.10	1.07	1.4	1.68
Amides	DMF	17.40	13.70	11.30	3.59	3.82	4.04

**Table 2:** HSP values of NBR vulcanizates.

NBR	δ_d_, MPa^1/2^	δ_p_, MPa^1/2^	δ_h_, MPa^1/2^	δ_t_, MPa^1/2^
NBR34	19.6	8.5	6.7	22.4
NBR39	19.6	9.6	6.7	22.8
NBR44	19.6	10.5	7.2	23.4

**Table 3:** The HSP values of IRM 903 and bio-diesel.

Fluids	δ_d_, MPa^1/2^	δ_p_, MPa^1/2^	δ_h_, MPa^1/2^	δ_t_, MPa^1/2^	Vmol, mL/mol
IRM 903	17.9	0.7	1.8	18.0	350
Bio-diesel	16.5	0.3	0.9	16.5	152
Ethanol	15.8	8.8	19.4	26.5	59

## Data Availability

The original contributions presented in the study are included in the article, further inquiries can be directed to the corresponding author.
